# Re-sequencing transgenic plants revealed rearrangements at T-DNA inserts, and integration of a short T-DNA fragment, but no increase of small mutations elsewhere

**DOI:** 10.1007/s00299-017-2098-z

**Published:** 2017-02-02

**Authors:** Henk J. Schouten, Henri vande Geest, Sofia Papadimitriou, Marian Bemer, Jan G. Schaart, Marinus J. M. Smulders, Gabino Sanchez Perez, Elio Schijlen

**Affiliations:** 1 Plant Breeding, Wageningen University and Research, Droevendaalsesteeg 1, 6708 PB Wageningen, The Netherlands; 2 Business Unit Bioscience, Wageningen University and Research, Droevendaalsesteeg 1, 6708 PB Wageningen, The Netherlands

**Keywords:** *Agrobacterium tumefaciens*-mediated transformation, Mutation frequency, Next-generation sequencing, Molecular characterization, Splinter, *Arabidopsis thaliana*

## Abstract

**Key message:**

Transformation resulted in deletions and translocations at T-DNA inserts, but not in genome-wide small mutations. A tiny T-DNA splinter was detected that probably would remain undetected by conventional techniques.

**Abstract:**

We investigated to which extent *Agrobacterium tumefaciens*-mediated transformation is mutagenic, on top of inserting T-DNA. To prevent mutations due to in vitro propagation, we applied floral dip transformation of *Arabidopsis thaliana*. We re-sequenced the genomes of five primary transformants, and compared these to genomic sequences derived from a pool of four wild-type plants. By genome-wide comparisons, we identified ten small mutations in the genomes of the five transgenic plants, not correlated to the positions or number of T-DNA inserts. This mutation frequency is within the range of spontaneous mutations occurring during seed propagation in *A. thaliana*, as determined earlier. In addition, we detected small as well as large deletions specifically at the T-DNA insert sites. Furthermore, we detected partial T-DNA inserts, one of these a tiny 50-bp fragment originating from a central part of the T-DNA construct used, inserted into the plant genome without flanking other T-DNA. Because of its small size, we named this fragment a T-DNA splinter. As far as we know this is the first report of such a small T-DNA fragment insert in absence of any T-DNA border sequence. Finally, we found evidence for translocations from other chromosomes, flanking T-DNA inserts. In this study, we showed that next-generation sequencing (NGS) is a highly sensitive approach to detect T-DNA inserts in transgenic plants.

**Electronic supplementary material:**

The online version of this article (doi:10.1007/s00299-017-2098-z) contains supplementary material, which is available to authorized users.

## Introduction

Authorisation for import or cultivation of genetically modified (GM) plants requires detailed risk evaluations for food, feed and environmental safety. In general, these evaluations include molecular characterization. At genomic level, this comprises characterization of T-DNA and vector sequence, copy number of inserts, assessment of flanking genomic regions, endogenous host gene interruptions by the T-DNA insert, and evaluation of homology between inserted and junction sequence to genes known to encode toxins or allergens (EFSA 2011). Routinely these genomic characterisations are based on ‘classical’ molecular techniques such as Southern blotting for copy number analysis of insert and vector integrations, and PCR, sequencing, and genome walking to reveal the DNA sequence of both inserts and flanking genomic DNA sequences of the host plant.

Next-generation sequencing (NGS) enables fast and reliable re-sequencing of complete genomes at relatively low costs, offering possible good alternatives for conventional techniques. Several approaches using NGS data for this purpose have been described (Kovalic et al. 2012; Wahler et al. 2013; Yang et al. 2013; Zastrow-Hayes et al. 2015; Pauwels et al. 2015; Guttikonda et al. 2016).

Whole genome re-sequencing of GM plants does not only provide information about T-DNA inserts and their flanking DNA, but delivers additional genome-wide sequence information. This enables comparative genomics between genomes of GM versus the non-GM plants. Deviations in the GM plant genomes can be caused by the transformation process itself, or can be a consequence of somaclonal variation, i.e. spontaneous mutations occurred during tissue culture, regeneration and propagation of the GM plant. Several studies have investigated mutations in transgenic plants compared to their non-GM parental plants. However these studies always included an in vitro phase (Kawakatsu et al. 2013; Ming et al. 2008). Moreover, these authors ascribed the detected mutations to in vitro cultivation and regeneration, rather than to the transformation process itself, although they could not prove this. Here, we used the floral dip method (Clough and Bent 2008) for *Arabidopsis thaliana* transformation, which circumvents in vitro propagation and regeneration, thereby excluding mutations due to somaclonal variation.

Information about type and frequency of mutations in GM plants is relevant for several reasons: (1) *A. tumefaciens*-mediated transformation is frequently used for analysis of gene functions. Mutations can have severe phenotypic effects, and can lead to misinterpretation of the function of the introduced gene(s); (2) mutations or rearrangements elsewhere in the genome of introduced GM crops can have adverse effects; (3) even in case the T-DNA is not present anymore in the progeny, the non-intended mutations might still be present. This holds also for crops derived from new breeding techniques (e.g. CRISPR-Cas9, TALENs, and reverse breeding).

In this study, we describe genome-wide comparative analysis of transgenic versus wild-type *Arabidopsis* plants, focussing on mutation detection, and analysis of structural variation such as large deletions and translocations.

## Materials and methods

### Gene construct and *Agrobacterium* transformation

A 3.7-kb promoter region of the *A. thaliana* gene SAUR8 (AT2G16580) was amplified from Col-0 genomic DNA, and recombined into pDONR207. The entry vector was subsequently recombined with the binary destination vector pBGWFS7 (Online Resource 1) providing Basta resistance (Karimi et al. 2002). The size of the T-DNA was 8379 bp. The resulting vector was used for transformation of *A. tumefaciens* strain C58C1 using electroporation (Weigel and Glazebrook 2005).

### *Arabidopsis* transformation

*Arabidopsis thaliana* Col-0 seeds were sown in square pots and grown under greenhouse conditions until flower bud formation. Transformation was performed using the *Agrobacterium*-mediated floral dip method (Clough and Bent 2008). Subsequently, seeds were harvested from single plants, and sown separately on 1/2MS plates (pH 5.8), containing 9 g/l agar and 15 mg/l Basta (phosphinothricin). Five Basta resistant seedlings derived from one single transformed parental plant were selected for DNA extraction and sequencing. These plants were named At1 to At5.

Another plant from the same initial seed batch, not subjected to floral dip transformation, was used for seed harvest. Also these seeds were sown, and upcoming seedlings were grown under same conditions except for Basta selection. DNA of four of these progeny plants was extracted.

### DNA isolation, library preparation and sequencing

Genomic DNA was isolated using a CTAB-based DNA isolation method (Doyle and Doyle 1987). DNA of the four non-transformed seedlings was pooled at equal quantities per seedling. DNA samples were randomly sheared using a Covaris E210 sonicator. Sheared DNA fragments were used for preparation of individual indexed libraries, suitable for Illumina HiSeq sequencing, using the Illumina TruSeq Nano DNA LT Sample Preparation Kit. Quality control of final libraries was performed on an Agilent Bioanalyzer DNA100 chip, and concentrations were determined using a Qubit fluorometer (Life Technologies). Final libraries had average fragment peak sizes of 600–650 bp. Barcoded libraries were pooled and analysed by means of an Illumina HiSeq 2000 sequencer, using 2 × 100 nt paired-end sequencing. After completion of sequencing, reads were de-multiplexed and assigned to original samples using Casava 1.8.2 software. Sequence reads were deposited at European Nucleotide Archive study accession PRJEB12451.

In addition, DNA of transgenic plants At2 and At5 was used for PacBio SMRTbell library preparation according to the manufacture’s protocol (10 kb Template Preparation and Sequencing with Low-input DNA, Pacific Biosciences). Final SMRTbells were size selected on a 0.75% agarose gel using a Blue pippin device (Sage sciences) with 5 Kb as cutoff for minimal fragment size. SMRTbell libraries were loaded at 0.03 nM using eight SMRT cells per library, and sequenced on a PacBio RS-II machine using C4/P6 chemistry, one cell per well, stage start and 300-min movie times.

### Analysis of T-DNA insert positions

**Fig. 1:**
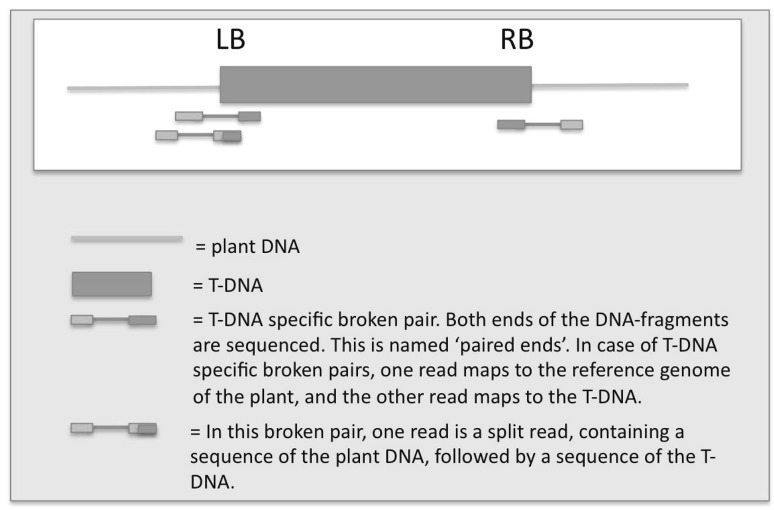
A cartoon representing ‘broken pairs’ and ‘split reads’. *LB* left border, *RB* right border

High-quality Illumina reads were mapped to the reference sequence of the *A. thaliana* Columbia Col-0 (Arabidopsis Genome Initiative 2000) genome version TAIR10, and to the vector and T-DNA as an additional, artificial chromosome. We specifically looked for broken read pairs and split reads (Fig. 1) that contained vector or T-DNA sequence, and mapped these to the reference genome for finding genomic positions of the T-DNA inserts. As the used gene construct contained a promoter from *A. thaliana*, we excluded this part of the T-DNA in the downstream analysis. Identified putative insert positions were verified manually, using visualization of read mappings by means of CLC genomics software, and applying heterozygous coverage of broken read pairs and split reads as criteria.

The existence of the small insert (‘splinter’) of plant At2 was verified by means of PCR using different combinations of the following primers: chr2F; TTGATGCTGCATTCCTGATCCGATTGT, chr2R; CCTATGTGATCTTTTGTGCTCCACCATCAC, Splinter cross border; AATGCCAGAAATGTCAATTTGATCAT.

PCR fragments of expected sizes were purified by gel electrophoresis and isolated using Qiagen minelute kit. Isolated fragments were quantified by Qubit. PCR fragments were pooled, using 10 ng per fragment, for PCR-free LT DNA library preparation following manufacturer’s instructions (Illumina). The obtained library was used for sequencing on a fraction of a MiSeq V2 flowcell with 2 × 250 nt paired-end reads.

### Detection of single nucleotide variants

We searched for single nucleotide variants (SNVs), comparing the sequences of the transformants and reference pool to the TAIR reference genome. Due to the large numbers of variants observed per line (average 5362 ± 123) and control pool (29,706) when compared to the TAIR reference genome, we concluded that the genomes of the *Arabidopsis* plants used deviated significantly from the published genome sequence of Columbia Col-0. For each transgenic plant, we executed a stringent variant calling compared to the reference genome TAIR (local variant coverage should be >10× minimal variant frequency 40%, ignoring non-specific regions), and compared these identified variants to the less stringently called variants (minimal variant frequency 10%) found within the non-transformed reference pool. We excluded common variants shared among transformants, as these SNVs were presumably inherited from the common parent, and not a result of transformation. There were 29 variants identified using criteria above. All genome positions of these identified SNVs across all individual transgenic plants as well as the wild-type plant pool were subjected to visual inspection. SNVs that appeared to be not unique, thus present in another plant but below thresholds used for automatic detection, were regarded as false and excluded. Eight SNVs remained that appeared to be specific for one transformant only, and completely absent in the other transformants and the analysed wild-type plants. In addition, we visually detected two more variants, close to T-DNA insert in plant At4, thereby increasing the final number of detected SNVs to 10.

All read mappings, variant callings, comparisons and filtering steps were performed using the alignment software Burrows-Wheeler Aligner (BWA) (Li and Durbin 2009), combined with command line scripts for downstream filtering, and CLC Genomics workbench 7.03 software for visualization of the putative variants.

### Detection of structural variants

Sequences of all transgenic as well as the non-GM plants were mapped to the reference genome of *A. thaliana*, and the complete vector sequence including the T-DNA, using BWA (Li and Durbin 2009) and the ΜΕΜ algorithm (Li 2013). BWA-MEM was run with seed length set to 19, bandwidth set to 25, and minimum length for re-seeding to 1.2. Additionally, BWA-MEM discarded seed matches that had 10 or more occurrences in the genome and gave as output all types of alignments, unique or multiple (option–a). This software provided Sequence Alignment/Map (SAM) files as output. These output files were converted into binary BAM files, using SAMtools (Li et al. 2009). Subsequently, DELLY v0.6.5 was run (Rausch et al. 2012). This software is able to call structural variants (SVs), including large genomic deletions, translocations, inversions and duplications, using information of broken pairs and split reads. The smallest detectable length of the called variations is around 300 nt. We used the tool at default settings, applying the multi-threading mode and specifying two memory threads per run, choosing as input a BAM file for one transgenic sample, the BAM file for the pooled sample of wild-type plants, and the *A. thaliana* TAIR10 reference genome with the vector and T-DNA sequence added.

We filtered for SVs that were specific for a transgenic plant, using an adjusted version of the python script somaticFilter.py that is provided with the DELLY package. For each SV type the minimum alternative allele frequency was set at 0.4. Results were further filtered using the following criteria: PASS filter in DELLY output, genotype call in both transgenic and wild-type non-GM sample, heterozygous genotype in transgenic plant and homozygous in non-GM plants, at least 10 broken pairs per SV, mapping quality higher than 50, and genotype quality higher than 30. Results were visually evaluated using CLC Main Workbench (CLC Bio, Qiagen).

To verify and reconstruct the identified T-DNA inserts and putative translocations, we produced PacBio sequencing data from the transgenic plants At2 and At5. For At2, 864,380 cleaned Pacbio reads with an average length of 4661 nt were aligned to the reference genome, using BLASR1.3.1.127046 as external application in CLC Bio software with the following settings: minMatch 14; -bestn 2; -minPctIdentity 0.70; -nCandidates 10. This mapping resulted in an average depth of 29× and >99.7% coverage of the genome. From plant At5 760,913 cleaned reads with an average length of 4721 nt were aligned to the reference genome, providing 26× sequencing depth, and >99.6% coverage.

## Results

### Re-sequencing and mapping

For floral dip transformation, immature floral buds of *A. thaliana* Col-0 plants were submerged in a suspension of transgenic *Agrobacterium tumefaciens* (Clough and Bent 2008). Seeds were harvested from single plants, and selected on Basta resistance, as the T-DNA included the *bar* gene conferring resistance to this herbicide. One of the parental plants produced five Basta resistant seedlings (At1 to At5), which were selected for genomic DNA extraction. In parallel, DNA from four pooled seedlings derived from a non-GM parent was isolated. DNA of both plant types was subjected to whole genome shotgun sequencing, using an Illumina HiSeq2000 system resulting in 2 × 101-nt-long paired-end sequence reads. High-quality reads were mapped to both the assembled sequence of the *A. thaliana* Columbia genome TAIR10 and the vector sequence including the T-DNA. For each genome the average coverage exceeded 25×, based on the mapped reads. A large fraction (>99.5%) of the reference genome of *A. thaliana* was covered after read mapping, indicating highly comparable datasets (Online Resource 2).

### Detection of single nucleotide variants (SNVs)

To detect mutations in the genomes of the transgenic plants, we focussed on the mapped reads from these plants, excluding read pairs with T-DNA sequences. Single nucleotide variants (SNVs) that were shared among transformants were excluded as these were inherited from the common parent, and SNVs in repetitive regions were also disregarded. As we used primary transformants, we selected for heterozygous polymorphisms only. Visual inspections of the resulting (29) heterozygous SNVs reduced the number to eight reliable SNVs, i.e. uniquely found in only one transgenic plant. During visual examination of T-DNA inserts, we identified two additional SNVs in proximity of a T-DNA insert in transformant At4. These SNVs were not identified using the approach described above, as they were present in read pairs containing T-DNA sequences (Table 1). The ten SNVs appeared in three transgenic plants, whereas we did not discover SNVs in the other two transgenic plants. Three SNVs occurred in an exon (Table 1). Two out of these resulted in a frame shift, which may disrupt the encoded protein.

**Table 1:** Single nucleotide variants (SNVs) detected in five transformants of *A. thaliana*

Plant	Region	Type	Reference	Allele	Length difference	Within gene	Exon/ Intron	Zygosity
At1	Chr2: 11,595,707–11,595,709	Deletion	GTG	–	3	AT2G27130	Exon	Heterozygous
At1	Chr2: 18,967,242.–18,967,245	Deletion	TTCC	–	4	–	–	Heterozygous
At1	Chr5: 23,122,680–23,122,684	Deletion	GGGTA	–	5	AT5G57120	Exon	Heterozygous
At1	Chr5: 24,549,125	Deletion	C	–	0	AT5G60990	Exon	Heterozygous
At3	Chr5: 5,539,059	SNP	A	T	0	AT5G16850	Intron	Heterozygous
At4	Chr2: 4,023,449	SNP	G	C	0	–	–	Heterozygous
At4	Chr3: 45,791	SNP	A	C	0	–		Heterozygous
At4	Chr3: 45,880	SNP	A	T	0	–		Heterozygous
At4	Chr4: 652,049–652,050	Deletion	AC	–	2	–	–	Heterozygous
At4	Chr5: 18,641,150–18,641,153	Deletion	GTAG	–	4	–	–	Heterozygous

Note that no SNVs were found in At2 and At5

### Localizing T-DNA inserts

To detect T-DNA inserts, we specifically looked for ‘broken pairs’, i.e. read pairs of which one read mapped to the plant genome whereas the other read mapped to either T-DNA or vector backbone (Fig. 1). We also focussed on single reads of which one part of the read mapped to the plant genome whereas another part mapped to T-DNA or backbone. These identified reads were called ‘split reads’ (Fig. 1 and Online Resource 3).

For each transgenic plant we selected broken pairs and split reads and mapped these back to the reference genome to find the chromosomal positions of T-DNA inserts. Identified putative insert positions were verified manually, using heterozygous coverage of broken read pairs and split reads as criteria, and applying visualization of read mappings using CLC Genomics Workbench. A total number of 12 inserts were identified in the five transformants. Transformant At5 contained only one T-DNA insert, all other transformants appeared to contain multiple (two to four) heterozygous T-DNA inserts (Table 2). Online Resource 3 provides an illustration of split reads from one plant mapped to the T-DNA, indicating the presence of T-DNA inserts at different sites within the genome of this plant. Multiple inserts clearly hampered assembly and reconstruction of the individual T-DNA inserts. Indications for inverted T-DNA repeats were found in two transformants (Table 2). Furthermore, six out of the 12 identified inserts were located in an open reading frame, thereby possibly interfering with the respective gene functions.

**Table 2:** Putative insert sites of T-DNA in the five *A. thaliana* transformants

Plant	Chr.	Position(s) in the reference genome	gDNA flanking the insert	Inserted T-DNA: positions on T-DNA vector	Orientation of T-DNA	Estimated insert size (bp)	Deletion in plant DNA at insert site (bp)	Number of broken pairs	Number of split reads	ORFs	Comment
At1	1 5	25,247,865 8,704,528	Chr1 at one side, Chr5 at the other side	LB—bar gene: 260–853	**−**	At least 594	18	72	9		Putative translocation of part of Chr1 into Chr5 next to T-DNA insertion site or part of Chr5 into Chr1. Exact size and type is unclear
At1	3	1,735,974–1,738,367	Both sides	LB—GUS gene: 252–7262	**–**	7011	2393	59	7	AT3G05830 (exon)	Almost complete T-DNA
At1	5	6,216,781	Right side	RB: –8244		At least 463	nd	24	1		
At1	5	6,221,171	Left side	LB: 242–		At least 442	nd	30	6	AT5G18660	
At2	1 2	3,607,701 12,291,248	Both sides	LB–RB 249–8,243	+	7996	4980	28	4	AAt1G10840 (exon)	Putative translocation of a distal part of Chr2 next to the T-DNA insertion site in Chr1^a^
At2	2	12,598,505–12,598,546	Both sides	Bar gene—Tnos (311–7967) + LB—Psaur (215–3914)		At least 11,355	34	64	9	AT2G29340	One full T-DNA insertion plus partial inverted repeat of T-DNA. Small (34 nt) deletion at insertion site^a^
At2	2	16,311,370–16,311,381	Both sides	*Gfp* gene 5890–5841	−	50	11	9	5	AT2G39080	‘Splinter’^a^
At3	2, 3	Chr2: 15,559,585 Chr3: 23,000,811	Both sides	LB–RB 250–8030	+	7781	0	26	4		Putative translocation of part of Chr2 into Chr3 next to T-DNA insertion site, or of part of Chr3 into Chr2, flanking the T-DNA
At3	5	14,491,644	Right side	Backbone: 13,206		At least 303	nd	29	3		
At4	2	272,511–272,527	Both sides	RB—Backbone: 8762–14,119		Unclear	16	42	0	AT2G01600	Putative inverted repeat of T-DNA, backbone vector sequence also included
At4	3	45,791–45,881	Both sides	LB–RB 1–8244	+	8244	90	45	8		
At5	1, 3	Chr1: 29,443,606 Chr3: 13,632,986	Left side	LB: 242	+	At least 4951	736,487	20	5		Putative translocation of a part of Chr3 into Chr1, next to T-DNA insertion. Large deletion in Chr1 starting at insertion. Upstream end of T-DNA insert. Also a large heterozygous deletion was found in Chr3 from the start of that chromosome up to position 182,859. (Fig. 4)^a^

*LB* left border, *RB* right border

^a^Verified by means of PacBio sequencing

### Detection of a ‘T-DNA splinter’

**Fig. 2:**
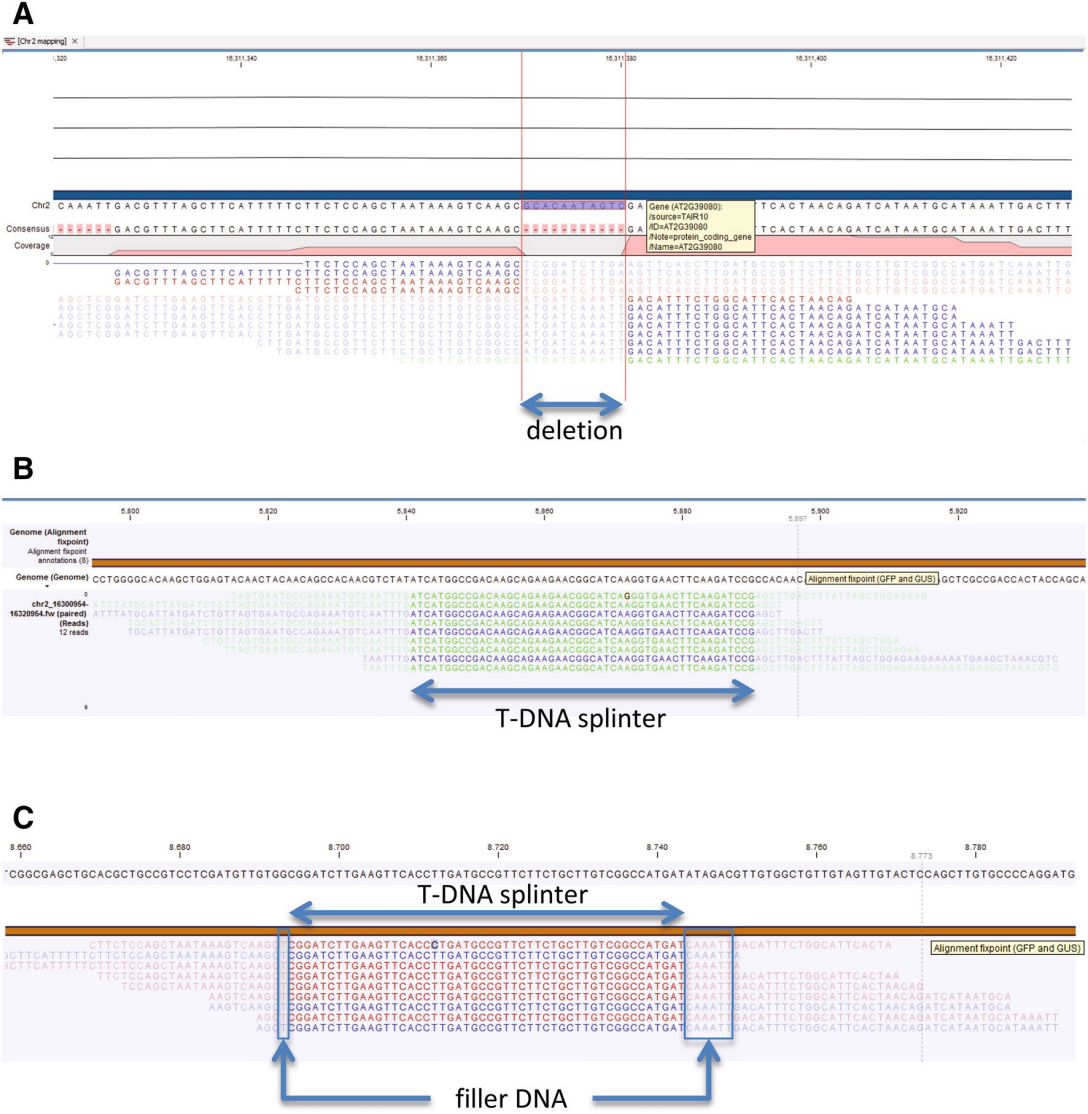
T-DNA splinter in the transgenic plant At2. Split reads composed of both plant and T-DNA derived sequences are represented by partial alignment (perfect aligned nucleotides in normal font, misaligned nucleotides displayed in transparent font). Reads were aligned to Chr2 as well as to the plasmid containing the gene construct and vector backbone. **a** Alignment to the reference genome of *A. thaliana* showing an 11 base pair deletion in Chr2 at the T-DNA insert site. **b** Split reads from At2 aligned to the plasmid sequence. The split reads perfectly aligned to a *gfp*-part in the T-DNA. **c** Reconstruction of the splinter insert, shown as read mapping to T-DNA. As the splinter was inserted in reverse orientation compared to the reference genome, the reverse complement sequences of the T-DNA reads are displayed. Filler DNA sequences are represented in boxes flanking both sides of the T-DNA splinter. Chromosomal DNA sequences flanking the insert are shown as transparent nucleotide sequences, and resemble the sequences flanking the deletion in A

Interestingly, one small insert was detected in transformant At2. This insert appeared to consist of a 50-base pairs (bp) fragment derived from the *gfp* gene encoding green fluorescent protein. This 50-bp fragment aligned perfectly to this *gfp* gene being part of the gene construct used for transformation, and located approximately in the middle of the T-DNA, far from the right and left border. As this insert encompassed only a small part of the T-DNA, we called it a ‘splinter’. We define a splinter as a small fragment of T-DNA or vector backbone, not coming from a border region, and being stably integrated in a host genome after transformation. The splinter was detected in this transformant only, not in any other transgenic *A. thaliana* plants. Furthermore, this splinter appeared to be heterozygous, confirming it was inserted into one chromosome during transformation. It appeared to be inserted in reverse orientation compared to the reference genome. Moreover, the splinter was detected by 12 split reads that mapped around position 16.311.370 of Chr. 2. Nine out of these 12 reads started in the plant genome, continued through the splinter *gfp* sequence, and resumed in the plant genome and, therefore, encompassed the complete 50-bp insert (Fig. 2). The remaining three split reads contained plant sequence and only a smaller part of the splinter, but confirmed the junction between plant genome and inserted sequence as revealed from the mentioned nine split reads. At the splinter insert site, the plant genome revealed an 11 bp deletion (Fig. 2A). Further scrutinizing the sequence information revealed 1-bp ‘filler DNA’ at the left side, and 6-bp ‘filler DNA’ at the right side of the T-DNA fragment (Fig. 2). This ‘filler DNA’ situated between the T-DNA fragment and plant gDNA contributed to a complete insert of 57 bp. The splinter was detected within an intron of gene AT2G39080.

To verify the presence of this splinter and its sequence, we designed primers on both flanking chromosomal sequences, as well as primers at the border of the insert (Online Resource 4). We performed PCR analysis to verify the splinter insert, using original isolated gDNA of At2. PCR results using two chromosomal primers on both sides of the insert confirmed the heterozygous status of the splinter, clearly showing two fragments. One fragment representing native plant DNA, another approximately 50 bp larger fragment also containing the inserted splinter (Online Resource 4). The amplicons that contained the splinter were subjected to sequencing. Results fully confirmed the presence, position and composition of the splinter, and the sequences shown in Fig. 2 for both homologous chromosomes.

**Fig. 3:**
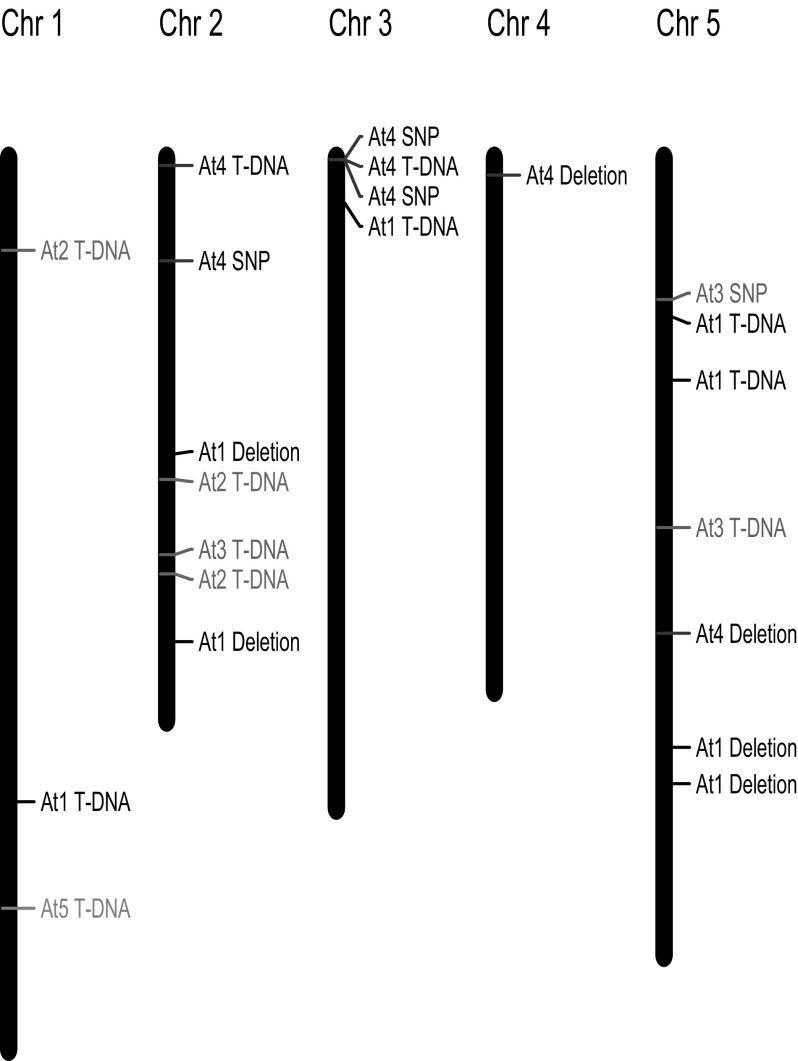
Position of T-DNA inserts and mutations as detected in the genomes of transgenic *A. thaliana* plants At1 through At5. Each transformant is represented by a *different colour*

Identified locations of the T-DNA inserts and SNVs in the genomes of the five transgenic plants are displayed in Fig. 3. Deletions at the insert sites (Table 2) are not included in Fig. 3. According to these results, there is no association between positions of detected small mutations and positions of the T-DNA inserts.

### Detection of structural variation and large deletions

Surprisingly, we found eight situations with a transition of plant chromosomal DNA into T-DNA at one end of the insert only, lacking the transition at the other insert side. However, T-DNA inserts in the genomes should be flanked at both sides by plant DNA, unless the T-DNA is at the very distal end of a chromosome, which was not observed (Fig. 2). This phenomenon might be caused by one side of the T-DNA ending in repetitive plant DNA, preventing mapping of reads to an unambiguous position. However, we did not find indications for this either. Alternatively, there could be a translocation of a DNA fragment originating from another chromosome, inserting at a double-strand break together with the T-DNA. Consequently, such translocation event would result in misleading identification of inserts in apparently two different chromosomes, showing one transition only between T-DNA and plant DNA per insert location. Therefore, we searched for putative structural variants (SVs), such as translocations in the transformants, using the software DELLY 0.65 (Rausch et al. 2012). We selected only heterozygous SVs that were specific for one transformant, and evaluated them visually. We detected that four out of 12 T-DNA inserts were flanked by sequences from two different chromosomes, in four different transformants (Table 2).

It was difficult to confirm the presence, nature and size of the putative translocations, using the current dataset of short reads. Therefore, we additionally produced PacBio sequencing data for two plants (At2 and At5), confirming the putative translocations besides T-DNA inserts in these plants.

**Fig. 4:**
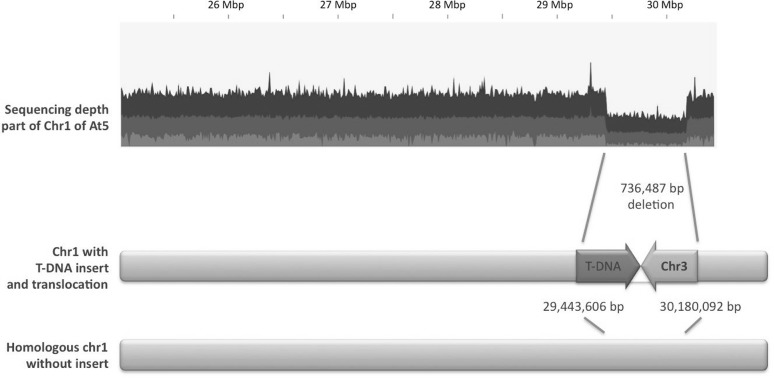
Large deletion in Chr1 found in transgenic plant At5. A clear drop in sequencing depth of mapped reads revealed a deletion of more than 736 kb. T-DNA was inserted at the start of this deletion. A distal part of Chr3 was inserted within this deletion region as well. The homologous chromosome of At5 remained intact, as illustrated by approximately 50% of overall coverage depth of mapped reads

At the majority of T-DNA insert sites, heterozygous deletions of plant genomic DNA were detected, ranging from 11 to 2.393 bp (Table 2). Remarkably, plant At5 contained a very large 736 Kb deletion downstream of Chr1 position 29,443,606 encompassing 214 genes (Fig. 4). Interestingly, at the end of this deletion, so upstream of Chr1 position 30,180,093, a heterozygous translocation of the *A. thaliana* genome was detected. This translocation originated from Chr 3, upstream of position 276,696 of Chr 3 (Fig. 4). Moreover, a heterozygous deletion of approximately 182 Kb at the beginning of Chr 3 was evident in this plant.

## Discussion

### Genome-wide small mutations after *A. tumefaciens*-mediated transformation

In our floral dip-mediated transgenic *Arabidopsis* plants, we detected an average of two small mutations compared to their common parent, disregarding the insert sites. This frequency of small mutations (2.0 ± 2.3 mutations per plant) is not significantly different from the frequency of 2.3 in seed-propagated plants without transformation (Ossowski et al. 2010). Further, we did not find a relationship between the positions of the T-DNA inserts and the small mutations, or a correlation between the number to T-DNA inserts per plant and the mutation frequencies of these plants. These results indicate that *A. tumefaciens*-mediated transformation, using floral dip, is not causing small mutations in the plant genome, disregarding the insert sites themselves, in spite of the possible stress caused by selection for resistance to the herbicide Basta.

### Mutations during tissue culture (somaclonal variation)

Previous studies have compared parental lines to transgenic plants obtained by in vitro propagation, *Agrobacterium*-mediated transformation and regeneration (Kawakatsu et al. 2013; Jiang et al. 2011; Miyao et al. 2012; Sabot et al. 2011). In these studies, the mutation rate was ~250 times higher than the base substitution frequency observed in sexually propagated plants. It has been suggested that this difference is due to somaclonal variation during in vitro culture, including activity of retrotransposons (Müller et al. 1990). As the genetic modification process usually includes a tissue or cell culture phase and a regeneration phase, mutations detected in GM plants compared to their parental plants are far more likely to have been caused by in vitro propagation than by the transformation itself.

### Deletions and translocations at the T-DNA insert sites

Our analyses of T-DNA insert sites in *A. thaliana* have clearly shown that genomic DNA was deleted in the majority of T-DNA insert sites (Table 1). These deletions were usually small but occasionally large deletions occurred affecting several or multiple genes. Both T-DNA inserts and genomic deletions were heterozygous, and the homologous chromosome still contained copies of the intact genes. However, in progeny homozygous for the T-DNA, the deletion or disruption of genes may have adverse effects. Potentially, this could result in decreased fitness or lack of progeny homozygous for this deletion.

In four cases, we detected putative translocations that were flanking T-DNA inserts. These translocated fragments originated from different chromosomes. It appeared difficult to detect reliably structural variants when using Illumina paired-end reads from relatively small DNA fragments with an insert size of approximately 600 bp. Therefore, we analysed the genomes of two plants, using PacBio sequencing that provided far longer reads of 4.7 kb on average. The PacBio data confirmed the putative translocations and deletions.

Translocations at T-DNA inserts have been described before (Curtis et al. 2009; Clark and Krysan 2010; Nacry et al. 1998; Tax and Vernon 2001). Remarkably, such T-DNA translocations have been reported only in transgenic *A. thaliana* when floral dip was applied. Possibly, the meiosis or zygote stage made floral dip more vulnerable for translocations compared to more common transformation methods using somatic tissue such as leaves or cotyledons.

We conclude that in case of floral dip in *A. thaliana*, the mutation frequency is high at the T-DNA insert sites, including large deletions and sometimes translocations.

### Natural variation

Cao et al. (2011) re-sequenced 80 strains of *A. thaliana*, representing the genetic diversity across the native range of the species in Eurasia. They identified nearly 5 million (4,902,039) SNPs across the 80 strains. This represents, on average, one SNP per 23 bp, taking all 80 strains into account. Most SNPs were not restricted to one strain only, but were found in at least two strains. More than 800,000 (810,467) small inserts/deletions (1–20 bp) were also detected in the 80 accessions (one-sixth of the number of SNPs), which is on the average one small indel per 140 bp. They detected at least 174,789 structural variants, of which 49% were detected in more than one strain. In the reference genome of *A. thaliana*, 31,189 transposable element inserts have been annotated. Of these transposable elements 80% showed evidence of being partially or completely absent from the genome of at least one of the 80 sequenced strains. This underlines the variability of these elements. Cao et al. (2011) discovered ‘drastic mutations’ in more than 6000 (6197) genes, probably blocking the biological functions of these genes. This highlights the enormous amount of standing genetic variation present in *A. thaliana*. Yogeeswaran et al. (2005) describe the high frequency of chromosomal rearrangements, including translocation and gene transpositions at an evolutionary scale, when comparing *A. thaliana* to the related species *A. lyrata*.

Kawakatsu et al. (2013) detected 196 mutations in a GM rice plant compared to its parent. Alignment of the non-GM parental line to the Nipponbare reference genome of rice, revealed > 500 times more polymorphisms between these two non-GM genomes.

This underlines that the frequencies of small mutations, (large) deletions and translocations, accumulated during evolution in plants species and used in conventional breeding programs, is multiple orders of magnitude larger than the frequencies of such mutations and structural variation caused by *A. tumefaciens*-mediated transformation, even when taking into account that at T-DNA insert sites, deletions in the plant genome are common, according to our study.

Schnell et al. (2014) reviewed insertional effects in GM plants such as deletions and rearrangements. They compared these with genomic changes occurring spontaneously in non-GM plants or during conventional breeding, such as deletions, translocations with double-strand breaks by non-homologous end-joining, and the intracellular transfer of organelle DNA. They concluded that changes at T-DNA sites are similar to changes occurring in non-GM plants.

### Splinter

We detected and confirmed the presence of one splinter, originating from the T-DNA used during transformation. This splinter was a 50-bp fragment from the *gfp* gene, derived from the middle part of the T-DNA, at more than 2 kb distance from both borders (Online Resource 4). As far as we know, this is the first report on occurrence of a ‘splinter’ in a transgenic plant. The coding region of a full *gfp* gene is ~717 bp. It is unlikely that the 50-bp insert will result into a functional peptide. In our case, the 50-bp fragment was inserted into an intron. No change in the coded protein was predicted, as the splinter will be spliced out, together with the native intron, according to gene prediction software. As a splinter is not a complete gene, it probably does not have a phenotypic effect that differs from commonly occurring mutations such as small indels.

We currently do not know what caused the insert of a splinter from the T-DNA, whether it is related to the floral dip transformation method used, and it is difficult to estimate the frequency of splinters in GM plants. Splinters, whenever they occur, may be lost in the process of producing seed-propagated GM crops or when the transgenic trait/locus is crossed into other varieties, because splinters, as all other T-DNA inserts, are present in heterozygous form in the primary transformant. During backcrossing of the transformant, approximately 50% of the progeny will not inherit the splinter. Repeated backcrossing will further reduce the probability of its presence. In case the splinter is genetically linked to the full T-DNA insert (so present on the same chromosome as the full T-DNA insert), and progeny plants are selected for presence of the GM trait, the likelihood of presence of the splinter will be higher than 50%. Conversely, if the splinter is present on the homologous chromosome compared to the full T-DNA (so in repulsion phase) the inheritance will be lower than 50% per generation. The essence, however, is that one should be able to detect and monitor splinters.

### NGS for detection of T-DNA inserts

NGS is a more sensitive technique for detection of T-DNA inserts in GM plants than Southern blotting. Also small partial T-DNA inserts (splinters) that may be overlooked by Southern blotting and PCR, can be detected when using NGS. Further, NGS can thus be used for revealing the flanking DNA of T-DNA inserts. Subsequently, the sequence of the flanking DNA can also be used for positioning of T-DNA inserts in the genome, in case a high-quality reference genome sequence is available for the plant species.

NGS is a useful technique for revealing flanking DNA of inserts, more efficient than genome walking. However, we had difficulties in assembling short Illiumina reads for characterisation of the full inserts, due to multiple inserts per plant, and the short-read sequencing combined with small library insert sizes used for paired-end sequencing. PacBio appeared to be helpful for this assembly.

#### Author contribution statement

HJS conceived and designed the research. JGS conducted DNA isolations. All NGS experiments were supervised by ES. HG, SP and ES performed the bioinformatics analysis, under supervision of GSP, and provided figures and tables. HS wrote the manuscript. JGS, MJMS and ES edited the manuscript. All authors read and approved the manuscript.

## Electronic supplementary material

Below is the link to the electronic supplementary material.

Supplementary material 1 (DOCX 377 KB)
