# Supplementary material for: Re-sequencing transgenic plants revealed rearrangements at T-DNA inserts, and integration of a short T-DNA fragment, but no increase of small mutations elsewhere
Source: Plant Cell Rep. 2017 Feb 2;36(3):493–504. doi: 10.1007/s00299-017-2098-z (PMC5316556; doi:10.1007/s00299-017-2098-z)
Supplement: Supplementary file 1 — Supplementary material 1 (DOCX 377 KB) [file 299_2017_2098_MOESM1_ESM.docx]

# Online Resource

**Online Resource 1.** Map of the vector and construct used for transformation of *A. thaliana*.

**Online Resource 2.** Number of reads and coverage per *A. thaliana* transformant At1 to At5, and the non-transformed reference pool.

**Online Resource 3.** Reads of transformant At1, mapped to the left end of the T-DNA (black sequence at top). The normal font refers to T-DNA, and the transparent font to genomic DNA of the host. Reads of intact mapped read pairs are in blue, reads of broken pairs in red. The figure illustrates that T-DNA has been inserted at three different loci in this plant.

**Online Resource 4.** Verification of the splinter in *A. thaliana* transformant 2 (At2); **A.** Used primer sequences; **B.** Graphical representation of primer positions on chromosome 2 containing the splinter; **C.** Digital Agilent Bioanalyzer gel image of obtained PCR fragments. The third lane shows both the amplified fragments with and without the splinter. These two fragments and the PCR fragment shown in the second lane were sequenced, and fully confirmed the DNA sequences of the splinter, flanking DNA, and of the homologous native locus, shown in Figure 2.


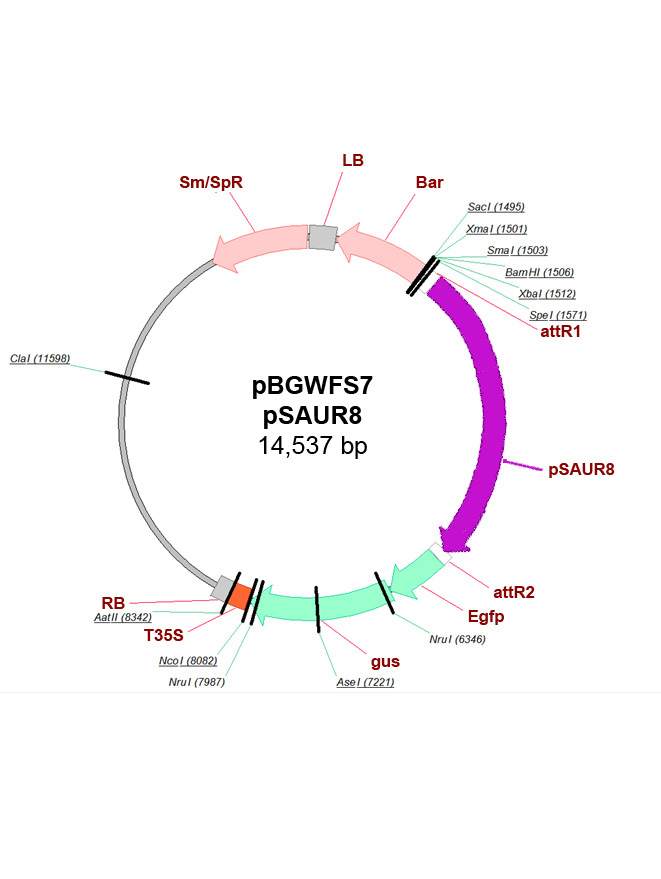
**Online Resource 1.** Map of vector and construct used for transformation of *A. thaliana*. 1-270: left border (LB) and surrounding DNA; 286-1400: *Bar* gene; 1631-5305: pSAUR promoter; 5329-5328: recombination site; 5377-7914: *gfp*; 7917-8179: Tnos; 8180-8325 : right border (RB) and surrounding DNA.

**Online Resource 2.** Number of reads and coverage per *A. thaliana* transformant At1 to At5, and the non-transformed reference pool.

| **Sample ID** | **Number of sequence reads** | **Number of reads after trimming** | **Read Length (nt)** | **% mapped reads** | **Average Coverage** |
| --- | --- | --- | --- | --- | --- |
| **At1** | 61683376 | 59480648 | 98.1 | 99.6% | 48.53 X |
| **At2** | 33049036 | 32103568 | 98.4 | 99.5% | 26.25 X |
| **At3** | 31968584 | 30902759 | 98.2 | 99.8% | 25.29 X |
| **At4** | 56915326 | 54937185 | 98.1 | 99.5% | 44.77 X |
| **At5** | 45006154 | 43359470 | 98.1 | 99.7% | 35.40 X |
| **WT1-4pool** | 158153422 | 152300479 | 98.1 | 99.5% | 124.1 X |


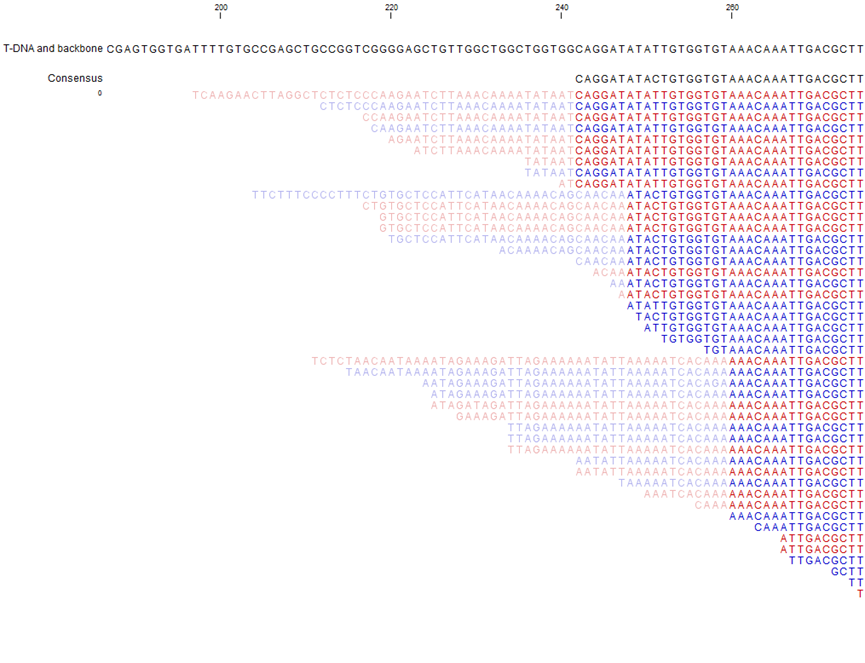
**Online Resource** **3.** Reads of transformant At1, mapped to the left end of the T-DNA (black sequence at top). The normal font refers to T-DNA, and the transparent font to genomic DNA of the host. Reads of intact mapped read pairs are in blue, reads of broken pairs in red. The figure illustrates that the T-DNA has been inserted into three different loci in this plant. This is consistent with Table 1. The expected ends of the T-DNA-inserts in the left border (LB), are at position 242. The detected ends appeared at positions 242, 248, and 260, illustrating that 0 to 18 bp have been truncated from the LB-end of the T-DNAs.

**A.**

| Primer name | Primer sequence |
| --- | --- |
| chr2 F | TTGATGCTGCATTCCTGATCCGATTGT |
| chr2 R | CCTATGTGATCTTTTGTGCTCCACCATCAC |
| splinter cross border (scb) | AATGCCAGAAATGTCAATTTGATCAT |

**B.**

**C.**

**Online Resource 4.** PCRs for verification of the presence of the splinter in *A. thaliana* transformant 2 (At2); **A.** The primer sequences; **B.** A cartoon showing the positions of the primers on the chromosome containing the splinter; **C.** Digital representation of the observed amplicons after PCR, using Agilent Bioanalyzer. The third lane shows both the amplicon without the splinter, and the amplicon containing the splinter. These two amplicons and the amplicon in the second lane were sequenced, and fully confirmed the DNA sequences of the splinter, of the flanking DNA, and of the homologous native locus, shown in Figure 2.
